# Nanophysical Antimicrobial Strategies: A Rational Deployment of Nanomaterials and Physical Stimulations in Combating Bacterial Infections

**DOI:** 10.1002/advs.202105252

**Published:** 2022-01-27

**Authors:** Bingqing Jia, Xuancheng Du, Weijie Wang, Yuanyuan Qu, Xiangdong Liu, Mingwen Zhao, Weifeng Li, Yong‐Qiang Li

**Affiliations:** ^1^ Institute of Advanced Interdisciplinary Science School of Physics Shandong University Jinan 250100 China; ^2^ Suzhou Research Institute Shandong University Suzhou 215123 China

**Keywords:** bacterial infections, drug‐resistance, nanomaterials, phototherapy, physical stimulations, sonotherapy

## Abstract

The emergence of bacterial resistance due to the evolution of microbes under antibiotic selection pressure, and their ability to form biofilm, has necessitated the development of alternative antimicrobial therapeutics. Physical stimulation, as a powerful antimicrobial method to disrupt microbial structure, has been widely used in food and industrial sterilization. With advances in nanotechnology, nanophysical antimicrobial strategies (NPAS) have provided unprecedented opportunities to treat antibiotic‐resistant infections, via a combination of nanomaterials and physical stimulations. In this review, NPAS are categorized according to the modes of their physical stimulation, which include mechanical, optical, magnetic, acoustic, and electrical signals. The biomedical applications of NPAS in combating bacterial infections are systematically introduced, with a focus on their design and antimicrobial mechanisms. Current challenges and further perspectives of NPAS in the clinical treatment of bacterial infections are also summarized and discussed to highlight their potential use in clinical settings. The authors hope that this review will attract more researchers to further advance the promising field of NPAS, and provide new insights for designing powerful strategies to combat bacterial resistance.

## Graphical abstract

Nanophysical antimicrobial strategies, the “beautiful and incredible” encounter between nanomaterials and physical stimulations, and their applications, as well as current challenges and further perspectives in combating bacterial infections, are systematically summarized and discussed. This review is anticipated to provide new insights into the design of powerful weapons for arduous campaigns against bacterial resistance.

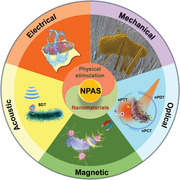

## 1 Introduction

Bacterial infections caused by the invasion of pathogenic bacteria into a susceptible host, pose a serious global challenge that threatens public health and causes heavy economic burdens.^[^ ^1^ ^]^ To survive in a world teeming with bacteria, extensive efforts have been made to develop powerful strategies to combat bacterial infections. The discovery of antibiotics in the twentieth century marked a new era of antibacterial campaigns and provided a transformative advantage in the fight against bacterial infections, not only because of their highly effective bactericidal ability but also because of their low toxicity to host cells.^[^ ^2^ ^]^ In the past few decades, antibiotics have become the first‐line antibacterial therapeutic modality and made great contributions to expanding life span and rewriting the history of public health. However, the injudicious use and application of antibiotics have led to the development of antibiotic tolerance and resistance.^[^ ^3^ ^]^ A variety of drug‐resistant bacteria, including methicillin‐resistant *Staphylococcus aureus* (MRSA), vancomycin‐resistant *S. aureus*, and carbapenem‐resistant *Enterobacteriaceae*, have emerged and are spreading at alarming rates worldwide, rendering antibiotics no longer a panacea for bacterial infections.^[^ ^4^ ^]^ These resistant bacteria are responsible for various community and hospital‐acquired infectious diseases, such as sepsis, meningitis, and pericarditis, thus becoming an extremely severe problem in the clinic.^[^ ^5^ ^]^ A recent World Health Organization (WHO) report suggested that resistant bacteria killed approximately 700 000 people every year globally, and this number is predicted to increase to 10 million by 2050, which will exceed the total deaths caused by cancer (8.2 million per year).^[^ ^6^ ^]^ This indicates that bacterial antibiotic resistance has been on a rise, calling for new antimicrobial drugs.

To better deal with resistant bacteria and formulate new powerful antibacterial strategies, it is necessary to understand the mechanisms underlying antibiotic resistance.^[^ ^7^ ^]^ In principle, the mechanisms underlying the emergence of bacterial antibiotic resistance mainly involve two aspects: the evolution of bacteria under the selection pressure of antibiotics and the formation of bacterial biofilms.^[^ ^8^ ^]^ It is well‐known that bacteria continuously mutate and evolve during their rapid reproduction, and the long‐term misuse of antibiotics expedites this mutational and evolutionary process.^[^ ^9^ ^]^ Under continuous antibiotic selection pressure, resistance acquisition occurs inevitably in several bacteria via specific biochemical routes, including modification of antibiotic target site, decreased penetration or promotion of the efflux of antibiotics, and hydrolase‐based antibiotic destruction.^[^ ^10^ ^]^ Apart from bacterial evolution, the formation of bacterial biofilms is another important mechanism for antibiotic resistance.^[^ ^11^ ^]^ Bacterial biofilms are densely packed bacterial communities that adhere to the surface of materials or tissues and are embedded in self‐produced extracellular polymeric substances (EPS).^[^ ^12^ ^]^ The EPS shield the bacteria from antibiotics and host innate immune cells, and provide a confined space to generate resistant bacterial strains by facilitating hypermutability and significantly increased rates of horizontal antibiotic‐resistant gene transfer.^[^ ^13^ ^]^

Faced with the grave threat of antibiotic resistance, researchers have pinned their hopes on the discovery of novel antibiotics that can circumvent the mechanisms of bacterial resistance.^[^ ^14^ ^]^ Unfortunately, the rate of discovery of new antibiotics is far behind that of the increase in bacterial resistance, and very few antibiotics have been developed in the past 30 years, heralding the arrival of the post‐antibiotic era.^[^ ^15^ ^]^ To deal with this situation, novel antimicrobial agents, such as natural antimicrobial compounds, bacteriophages, and antimicrobial peptides (AMPs) have been explored to treat bacterial infections.^[^ ^16^ ^]^ Although these alternative antimicrobial agents have shown promise in the treatment of antibiotic‐resistant infections, the relatively low antimicrobial activity of natural compounds, the narrow‐spectrum bactericidal ability of bacteriophages, and the limited stability of AMPs under physiological conditions hinder their clinical translation.^[^ ^17^ ^]^ Thus, the need for alternative antimicrobial strategies continues.

A physical approach based on the stimulation of physical signals is considered a promising therapeutic modality for bacterial antibiotic resistance.^[^ ^18^ ^]^ Unlike antibiotics that act on and react with a specific target without destroying bacterial morphology, physical stimulation implements alternative mechanisms that act on multiple targets to disrupt bacterial structure (e.g., cell wall and membrane) by mechanical stretching or by interfering with microbial metabolism and respiration.^[^ ^19^ ^]^ As a result, physical antimicrobial approaches can not only decrease the emergence of microbial resistance greatly, but can also kill resistant bacteria. Meanwhile, the rapid development of nanotechnology has resulted in brighter prospects for the application of physical antimicrobial methods.^[^ ^20^ ^]^ Compared with traditional large‐scale materials, nanomaterials have unique physical properties (e.g., optical, mechanical, thermal, and electromagnetic) because of their special size, high specific surface area, and biofunctionality (e.g., blood retention, tissue permeability, and bacteria targeting) after modification.^[^ ^21^ ^]^ These unique characteristics of nanomaterials greatly reinforce their efficacy and extend the application range of physical antimicrobial approaches, especially in the biomedical field. Moreover, by adjusting its composition and structure, one nanomaterial can respond to a variety of physical stimuli, thus acting as an excellent toolbox to combine and exploit the complementary advantages of multiple physical antimicrobial modalities, enabling better treatment of bacterial infections.^[^ ^22^ ^]^

**Figure 1:**
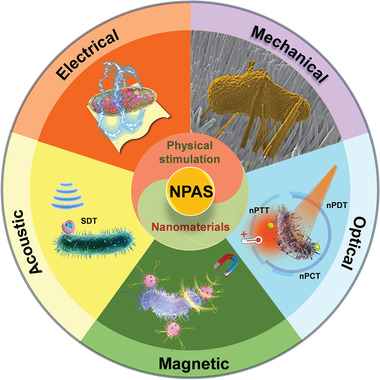
Typical NPAS categorized according to their mode of physical stimulation, including mechanical, optical, magnetic, acoustic, and electrical signals. Reproduced with permission.^[^ ^28^ ^]^ Copyright 2020, Nature Publishing Group.

Benefiting from the superior advantages of physical antimicrobial approaches and nanomaterials, nanophysical antimicrobial strategies (NPAS), the “beautiful and incredible” encounters between nanomaterials and physical stimulation, have become essential and well‐traveled investigation fields, and various exquisite NPAS has been designed for bacterial infection treatment. To provide a timely summary of this rapidly growing field, this review elaborates and categorizes the recently developed key advanced NPAS that can potentially revolutionize future antibacterial therapy, according to their mode of physical stimulation, which includes mechanical, optical, magnetic, acoustic, and electrical signals (**Figure** **1**). The biomedical applications of NPAS in combating bacterial infections are systematically introduced, with a focus on their design ideas and antimicrobial mechanisms. The current challenges and future perspectives of NPAS for the treatment of clinical bacterial infections are also presented and discussed. We envision that this review will provide novel insights into the design of powerful weapons for arduous campaigns against bacterial resistance.

## 2 Mechanical Stimulation‐Based NPAS

Inspired by nature, researchers have found that specific nanogeometric surfaces possess unique anti‐biological fouling and antibacterial properties, which promote a new generation of antimicrobial strategies that eliminate microbes through mechanical stimulation.^[^ ^23^ ^]^ There is an inherent physical interaction between nanogeometry and bacterial extracellular organelles (e.g., flagella and pili), leading to enhanced bacterial attachment on nanogeometric surfaces.^[^ ^24^ ^]^ Moreover, nanogeometry can elicit mechanical forces upon contact with bacteria to destroy their structure and achieve bacterial elimination.^[^ ^25^ ^]^ This section summarizes antimicrobial nanogeometries from the perspective of their mechanical bactericidal mechanisms, including mechanical stretching and “nanoknife” cutting, so that researchers can apply them flexibly according to specific actual conditions.

### 2.1 Mechanical Stretching Mechanism

**Figure 2:**
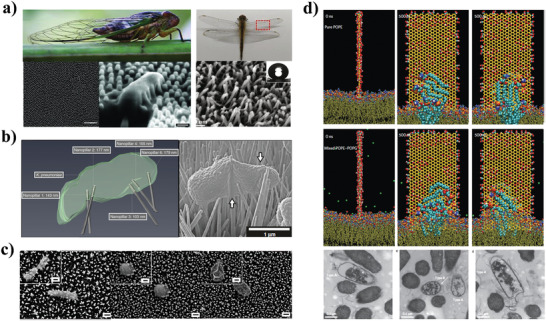
Mechanical stimulation‐based NPAS. a) Photograph of cicada (left) and dragonfly (right), and scanning electron microscopy (SEM) images of the upper surfaces of their wings. Reproduced with permission.^[^ ^26a^ ^]^ Copyright 2012, Wiley‐VCH (left). Reproduced with permission.^[^ ^26b^ ^]^ Copyright 2020, Elsevier (right). b) Nanopillar‐induced envelope deformation in *Klebsiella pneumoniae*. Reproduced with permission.^[^ ^28^ ^]^ Copyright 2020, Nature Publishing Group. c) SEM images of *P. aeruginosa*, *S. aureus*, and *B. subtilis* spores with destroyed structures attached on the surface of black silicon. Reproduced with permission.^[^ ^29^ ^]^ Copyright 2013, Nature Publishing Group. d) Destructive extraction of phospholipids from *E. coli* membranes based on molecular dynamics simulations and transmission electron microscopy. Reproduced with permission.^[^ ^39^ ^]^ Copyright 2013, Nature Publishing Group.

In nature, cicada and dragonfly wings, whose surfaces consist of specific nanogeometry, have been found to possess inherent antibacterial properties (**Figure** **2**a).^[^ ^26^ ^]^ To explore the mechanism behind this phenomenon, researchers have conducted various systematic studies. In 2012, Ivanova and his colleagues found that the cicada wing surface consisted of an array of nanopillars and effectively killed *Pseudomonas aeruginosa* (*P. aeruginosa*) on direct contact, showing outstanding antimicrobial ability. Moreover, when the surface of the cicada wing was chemically modified, its antibacterial effect did not change, indicating that the mechanism was related to the physical nanogeometry (nanopillar) of the cicada wing surface. To better understand the antibacterial mechanism of nanopillars, Pogodin et al. established a model to investigate the interaction between bacterial membranes and nanopillars.^[^ ^27^ ^]^ The results showed that when bacteria were in contact with the nanopillars, the bacterial cell membrane was adsorbed and mechanically stretched by the nanopillar geometry, and bacterial rupture would occur if the tensile strength was sufficiently high. Jenkins et al. characterized the physiological and morphological effects of mimetic titanium nanopillars on bacteria, and they found that nanopillars induced deformation and penetration of both gram‐negative and gram‐positive bacterial cell envelopes, thereby inhibiting bacterial cell division and causing bacterial cell death (Figure 2b).^[^ ^28^ ^]^ These studies establish an understanding of the antibacterial mechanism of the nanopillar geometry and lay a solid foundation for further in‐depth research on nanogeometry‐based NPAS.

Based on the understanding of the principle of antimicrobial nanopillar geometry, Ivanova et al. used reactive ion etching to synthesize black silicon (bSi) nanopillar arrays with structural features similar to the surface nanogeometry of cicada wings.^[^ ^29^ ^]^ They found that the bSi nanopillars had obvious antibacterial effects on *P. aeruginosa* through mechanical stretching of the bacterial cell membrane (Figure 2c). However, the bSi nanopillar surface is quite fragile and can easily be damaged even by a human nail dragged across its surface.^[^ ^30^ ^]^ Therefore, a sharp boron‐doped diamond (BDD)‐coated bSi nanoneedle, called “black diamond,” was constructed as an alternative. The advantage of BDD coating is a more robust and stable nanogeometric structure that is less likely to be damaged by external factors, and can also enhance the bactericidal effects of nanoneedles, eliminating a higher percentage of bacterial cells through mechanical stretching.^[^ ^31^ ^]^ Apart from nanopillars and nanoneedles, other nanogeometric structures, such as nanowires and nanocones, were found to exhibit bactericidal capability through mechanical stretching. It should be pointed out that compared to gram‐negative bacteria, the mechanical stretching‐involved bactericidal mechanism shows relatively lower antimicrobial performance toward gram‐positive bacteria, such as *S. aureus*, *Bacillus subtilis* (*B. subtilis*), and *Pseudococcus maritimus*.^[^ ^32^ ^]^ This phenomenon is attributed to the thicker peptidoglycan structure outside gram‐positive bacteria that can withstand greater deformation stress, rendering bacteria resistant to damage by nanogeometric mechanical stretching.^[^ ^33^ ^]^ In addition, some key nanogeometric parameters, such as the height and diameter of the tip and base, periodicity and flexibility, and the elasticity of the cell membrane of different bacteria, are all key factors that need to be considered.^[^ ^34^ ^]^

### 2.2 “Nanoknife” Cutting Mechanism

In addition to the mechanical stretching mechanism, some nanogeometric structures with sharp edges have been reported to insert and cut the bacterial cell membrane, demonstrating a powerful antimicrobial effect called “nanoknife” cutting.^[^ ^35^ ^]^ Akhavan et al. studied the bacterial toxicity of graphene nanosheets deposited on a stainless steel substrate in the form of graphene nanowalls.^[^ ^36^ ^]^ The experimental results showed that the bacterial cell membrane was punctured by the sharp nano‐edges of the graphene nanosheet, causing irreversible cell damage and leakage of intracellular contents, finally resulting in the death of bacterial cells. More interestingly, gram‐negative *Escherichia coli* (*E. coli*), with an outer membrane, was found to be more tolerant than the gram‐positive *S. aureus*, without the outer membrane protection, to the invasion of graphene nanosheets. Therefore, in contrast to the bactericidal mechanism of mechanical stretching induced by the nanopillars discussed above, the damage caused by the sharp nanosheets is largely derived from mechanical puncture. Yu et al. further studied the interaction between bacteria and graphene oxide (GO) nanosheets and demonstrated that the size of the nanosheets was an influencing factor for their bactericidal effects.^[^ ^37^ ^]^ In detail, a small‐sized GO nanosheet can produce a stronger cell cutting effect on bacterial cells, while a larger‐sized GO nanosheet majorly works on the phenomenon of bacterial cell entrapment. Therefore, the size of the nanosheets needs special consideration in actual antimicrobial applications. In addition to graphene nanosheets, the sharp edges of titanium nanosheets can also insert into and cut cell membranes, thus exhibiting excellent antibacterial ability.^[^ ^38^ ^]^

Recently, studies have shown that the damage inflicted by graphene nanosheets on bacterial cells is not only due to the cutting effects on their cell membranes, but also due to the extraction and redirection of membrane lipids. Based on molecular dynamics simulations and experimental investigations, Tu et al. found that graphene nanosheets suspended above the membrane can effectively extract phospholipid molecules in the bacterial cell membrane onto its surface, resulting in the deformation or loss of the integrity of the bacterial cell membrane (Figure 2d).^[^ ^39^ ^]^ The strong attraction between graphene nanosheets and membrane lipids stems from the 2D structure of graphene, and the van der Waals attraction between the two also occupies an important role. After the lipids were extracted, the interaction between the graphene nanosheet and the hydrophobic ends of the lipids further promoted the reorientation of the lipids on the nanosheets. Recent studies have further demonstrated that the behavior of 2D nanosheets to extract lipids from lipid membranes is closely related to temperature. Through molecular dynamics simulations, Li et al. found that the lipid extraction behavior of boron nitride (BN) nanosheets was very sensitive to temperature.^[^ ^40^ ^]^ Although BN nanosheets can quickly bind to the lipid membrane, lipid extraction behavior is not exhibited at low temperatures. Further studies have shown that temperature‐dependent lipid extraction behavior is related to the phase transition temperature of the lipid membrane. When the ambient temperature dropped to the phase transition temperature, the transformation of the membrane from the disordered lipid phase to the ordered gel phase increases its strength, thereby hindering the lipid extraction process. In addition, it has been reported that the leakage of intercellular molecules is due to the local phase transition caused by the interaction between 2D nanomaterials and cell membranes, which may also be an important factor that contributes to bacterial lysis.^[^ ^41^ ^]^

Compared to antibiotics and antibacterial drugs that need to act on and react with a specific target, nongeometric structures with mechanical stretching and “nanoknife” cutting characteristics can effectively damage bacterial structures; they are a more advantageous and promising solution for the challenge of bacterial resistance.^[^ ^42^ ^]^ In actual antimicrobial applications, the structure‐activity relationship of nanogeometric materials needs to be systematically investigated to obtain better bactericidal and treatment performance.

## 3 Optical Stimulation‐Based NPAS

Optical stimulation, especially ultraviolet (UV) light, can directly inhibit DNA replication and growth of bacteria, and has been used as a powerful physical sterilization method for medical disinfection.^[^ ^43^ ^]^ With the development of nanotechnology, various optical stimulation‐based NPAS has recently been developed by exploiting the unique physical properties of nanomaterials. These unique properties overcome the shortcomings of traditional UV light disinfection (e.g., toxic side effects and poor bacterial targeting) and help achieve reinforced antimicrobial performance.^[^ ^44^ ^]^ In this section, three main optical stimulation‐based NPAS, namely nano‐photocatalytic, nano‐photodynamic, and nano‐photothermal therapy, are categorized and systematically introduced according to their inherent bactericidal mechanisms.

### 3.1 Nano‐Photocatalytic Therapy (nPCT)

**Figure 3:**
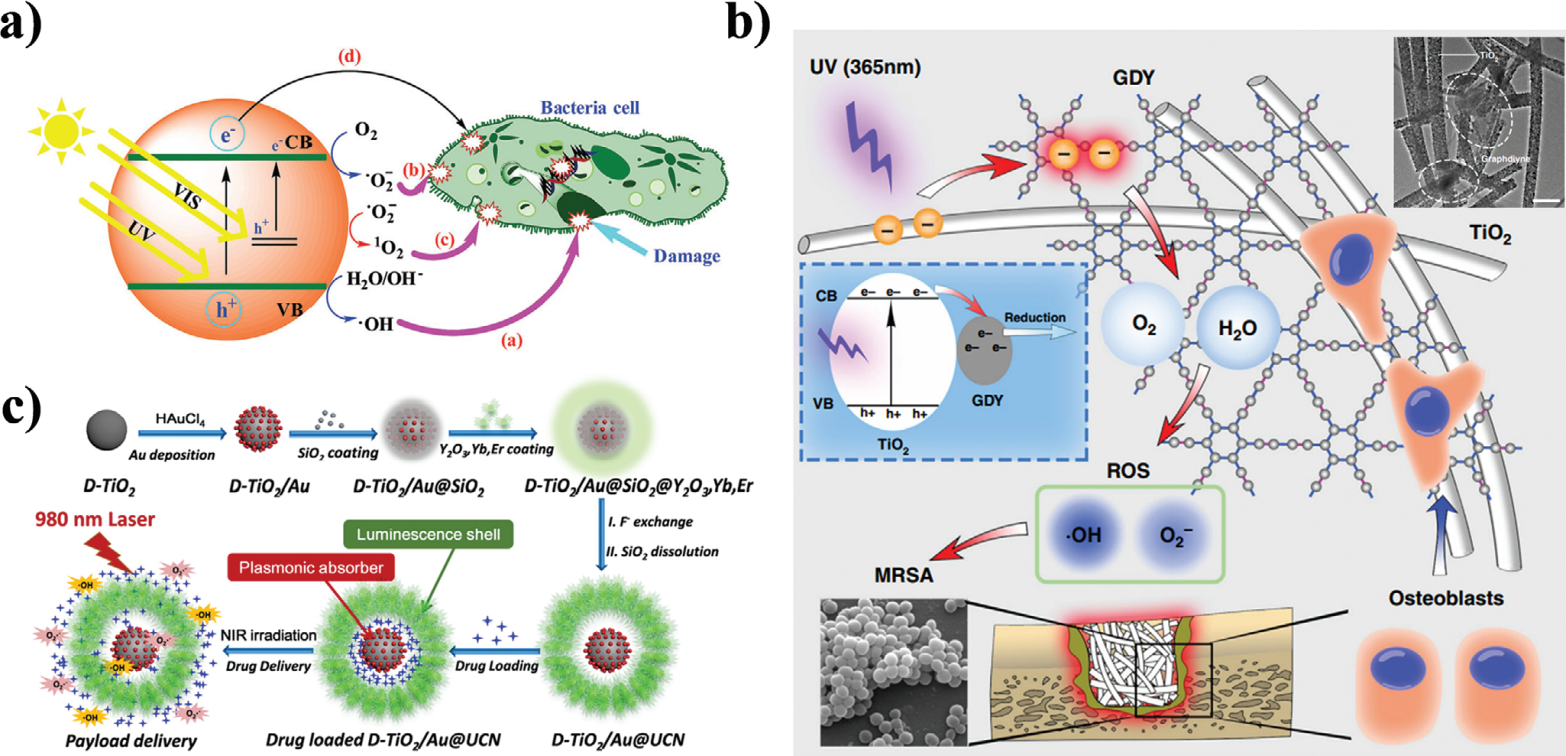
Antimicrobial nPCT. a) Schematic illustration of antibacterial nPCT. Reproduced with permission.^[^ ^47^ ^]^ Copyright 2017, Royal Society of Chemistry. b) Schematic of the dual function of TiO_2_/graphdiyne in orthopedic implant infection treatment under UV irradiation. Reproduced with permission.^[^ ^50^ ^]^ Copyright 2020, Nature Publishing Group. c) Construction of NIR light‐triggered photocatalytic nano‐system of D‐TiO2/Au@UCN for bacterial inactivation. Reproduced with permission.^[^ ^52^ ^]^ Copyright 2020, American Chemical Society.

Nano‐photocatalysis is generally defined as the catalysis of a photochemical reaction at the surface of semiconductor nanoparticles.^[^ ^45^ ^]^ In this process, light with energy greater than the band gap of semiconductor nanoparticles excites electrons from the valence band (VB) of semiconductor nanoparticles to the conduction band (CB), thus forming a pair of negatively charged free electrons and positively charged holes. The produced electrons and holes have strong reducing and oxidizing abilities, respectively, and can generate various reactive oxygen species (ROS), including hydroxyl radicals (∙OH), superoxide radicals (∙O_2_^−^), and singlet oxygen (^1^O_2_) at the surface of the semiconductor nanoparticles.^[^ ^46^ ^]^ These photogenerated ROS can cause the peroxidation of lipids in the outer cell wall and the disruption of cell organelles and DNA inside microbes, ultimately resulting in bacterial death (**Figure** **3**a).^[^ ^47^ ^]^ In recent years, a large number of photocatalytic nanomaterials have been designed, and nPCT has emerged as a promising modality for broad‐spectrum microbial inactivation.

As a classic photocatalytic nanomaterial, titanium dioxide (TiO_2_) nanoparticles have attracted much attention in the field of photocatalytic disinfection due to their good biocompatibility and stability.^[^ ^48^ ^]^ Studies have shown that the crystal phase of TiO_2_ nanomaterials has a significant influence on their photocatalytic activity. Among all the crystal phases (anatase, brookite, and folds), anatase TiO_2_ possesses the highest photocatalytic activity and has become the favored choice among TiO_2_ photocatalysts, due to its hydroxyl groups, larger band gap, higher surface area, and porosity.^[^ ^49^ ^]^ Recently, Wang et al. synthesized TiO_2_/graphdiyne (TiO_2_/GDY) nanofiber photocatalysts by changing the surface charge of GDY sheets and anatase TiO_2_ nanofibers and assembling GDY onto TiO_2_ nanofibers using electrostatic force (Figure 3b).^[^ ^50^ ^]^ For this TiO_2_/GDY nanofiber photocatalyst, the special *sp* and sp^2^ hybrid carbon atoms of GDY facilitated the transfer of photogenerated electrons from TiO_2_ to GDY under UV light irradiation, thus extending the lifespan of photogenerated electrons and greatly enhancing the photocatalytic effect of TiO_2_ to generate more ROS for bacterial killing.^[^ ^51^ ^]^ Furthermore, to overcome the limitations of UV light penetration depth in TiO_2_ photocatalytic reactions, many novel hybrid TiO_2_ photocatalytic nanoparticles have been developed. Recently, Xu et al. synthesized upconversion NaYF4 and Yb/Er nanocrystals co‐doped with Au nanoparticle‐deposited TiO_2_ nano‐photocatalysts, and constructed a near‐infrared (NIR) light‐triggered photocatalytic nanosystem (D‐TiO_2_/Au@UCN) for bacterial inactivation (Figure 3c).^[^ ^52^ ^]^ By matching the emission of upconversion nanocrystals with the surface plasmon resonance absorption of Au nanoparticles, this hybrid TiO_2_ nano‐photocatalyst enabled highly efficient ROS generation under NIR light irradiation, leading to bacterial elimination. In addition to their inherent antimicrobial activity, these hybrid TiO_2_ nanoparticles could effectively load bactericides to achieve synergistic bactericidal effects.

Apart from TiO_2_‐based nanoparticles, other semiconductor nano‐photocatalysts, especially those that can be activated under visible light, have been employed in antimicrobial nPCT. Jin et al. prepared caged iodine‐modified ZnO nanoparticles (I‐ZnO‐n), and demonstrated that iodine modification and cage nanostructures produced surface oxygen vacancy defects that were conducive to the separation of photogenerated electron‐hole pairs and significantly improved the photocatalytic antimicrobial performance of I‐ZnO‐n.^[^ ^53^ ^]^ The experimental results showed that I‐ZnO‐n possessed stronger absorption in visible light, and had better photocatalytic antibacterial activity against *E. coli* than ZnO nanoparticles did. Recently, visible‐light‐activated metal‐free graphitic carbon nitride g‐C_3_N_4_ photocatalysts have proved to exhibit attractive advantages due to their simple preparation method, good stability, and biocompatibility. Unfortunately, the antibacterial activity of g‐C_3_N_4_ is severely limited owing to its rapid carrier recombination and low specific surface area.^[^ ^54^ ^]^ Many strategies have been reported to solve these issues and improve the photocatalytic antibacterial efficiency of g‐C_3_N_4_ materials. The most used strategies are doping elements and compound modification to construct heterojunctions.^[^ ^55^ ^]^ The doping element can effectively change the band gap structure and improve light absorption efficiency. Moreover, defect sites are introduced in the lattice of g‐C_3_N_4_ to trap free electrons and holes, thereby prolonging the carrier lifetime and preventing recombination. Composite modification to form a heterojunction can transfer electrons or holes generated under light radiation to a more suitable CB or VB in the composite material to optimize the energy band structure and promote the generation of ROS. In addition, by adjusting the structure of g‐C_3_N_4_ or by introducing functional groups, the specific surface area and hydrophilicity of the material can be increased, thereby optimizing the charge transport distance and improving its photocatalytic efficiency.^[^ ^56^ ^]^ Recently, Wu et al. constructed a manganese dioxide (MnO_2_)/g‐C_3_N_4_ heterojunction, and explored its antibacterial properties.^[^ ^57^ ^]^ Since MnO_2_ could promote the transfer and separation of free charges and oxidize glutathione, which protects bacteria from oxidative stress, the MnO_2_/g‐C_3_N_4_ composite had an extremely high utilization efficiency of visible light and improved antimicrobial efficiency compared to that of g‐C_3_N_4_ alone. In addition to ZnO and g‐C_3_N_4_, other photocatalysts, such as molybdenum trioxide (MoO_3_), tungsten trioxide (WO_3_), and bismuth trioxide (Bi_2_O_3_) have also been synthesized and exploited in antimicrobial nPCT.^[^ ^58^ ^]^

### 3.2 Nano‐Photodynamic Therapy (nPDT)

Photodynamic therapy (PDT), a process that combines photosensitizers (PS) and light stimulation of an appropriate wavelength to induce oxidative damage in targeted cells, has been regarded as a promising therapeutic modality for the treatment of bacterial infections.^[^ ^59^ ^]^ During PDT, the ground state of PS is first changed to a short‐lived singlet excited state (^1^PS*) after absorbing a photon under light stimulation, and the ^1^PS* can then be changed to a long‐lived triplet state (^3^PS*) by intersystem crossing. The produced ^3^PS* can either transfer its electrons to surrounding substrates to generate radical ions (e.g., ∙OH and ∙O_2_^−^) via the Type I pathway, or transfer its energy to molecular oxygen to generate ^1^O_2_ via the Type II pathway.^[^ ^60^ ^]^ These generated radical ions and ^1^O_2_ can effectively destroy the protein, lipid, and genetic material of bacterial cells via oxidative damage, leading to bacterial death.^[^ ^61^ ^]^ However, PS tends to accumulate and quench in water, and its shortcomings of rapid metabolism and circulation in the body greatly limit its antimicrobial applications.^[^ ^62^ ^]^ In recent years, the prosperity of nanotechnology has provided strong support for the development of nPDT, and various antimicrobial nPDT systems have emerged to achieve reinforced bactericidal performance.

**Figure 4:**
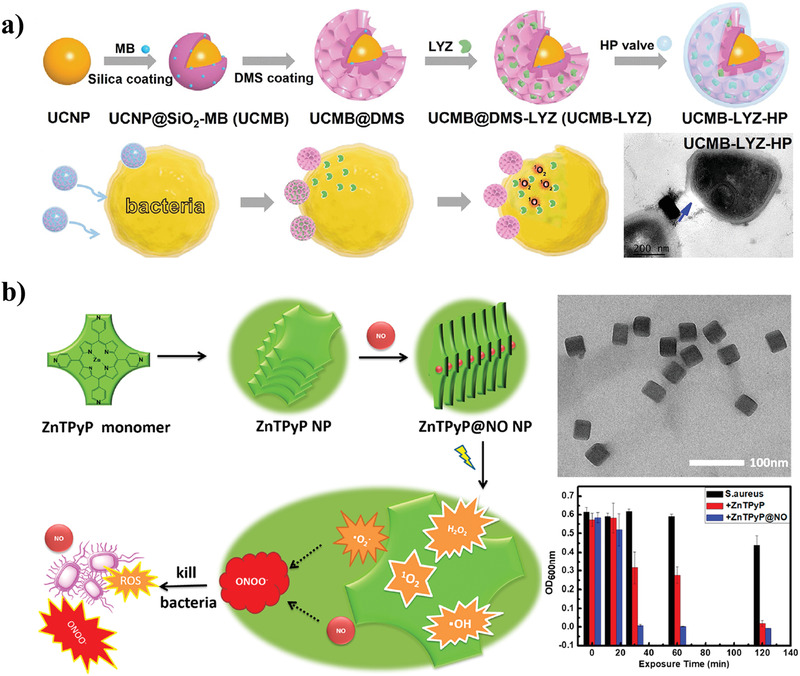
Antimicrobial nPDT. a) The fabrication process and proposed antimicrobial mechanism of the nanohybrid UCMB‐LYZ‐HP. Reproduced with permission.^[^ ^65^ ^]^ Copyright 2021, Wiley‐VCH. b) The preparation and antimicrobial activity of self‐assembled ZnTPyP@NO. Reproduced with permission.^[^ ^66^ ^]^ Copyright 2018, American Chemical Society.

Nanomaterials can be used as carriers of PS to improve their stability and blood circulation and achieve enhanced photodynamic antimicrobial effects. By covalently linking the PS of Chlorin e6 (Ce6) to iron oxide nanoparticles (Fe_3_O_4_ NPs) and further modifying them with bacterial species‐identifiable aptamers, our group reported a targeted antimicrobial nPDT system (Fe_3_O_4_‐Ce6‐Apt) for extracorporeal blood disinfection.^[^ ^63^ ^]^ Given the protective effect of Fe_3_O_4_ NPs on Ce6, this photodynamic Fe_3_O_4_‐Ce6‐Apt nanosystem enabled complete extracorporeal blood disinfection, and experiments demonstrated that the disinfected blood could be reused for mouse transfusion without inducing adverse reactions. In addition to Fe_3_O_4_ NPs, biocompatible silica nanoparticles have also been exploited to construct a powerful antimicrobial nPDT. By modifying polyelectrolyte‐coated silica nanoparticles with Ce6, we presented a novel bacteria‐activated nPDT system (SiO_2_/PAH‐Ce6).^[^ ^64^ ^]^ The ^1^O_2_ generation of Ce6 in SiO_2_/PAH‐Ce6 was quenched by the aggregation of Ce6 on silica nanoparticles to induce excited‐state quenching, and would be recovered by bacteria‐activated dissociation of polyelectrolyte‐Ce6 complexes from silica nanoparticles to achieve targeted PDT against MRSA. Recently, antimicrobial nPDT with deeper tissue penetration was constructed based on silica nanoparticles. Li et al. encapsulated lanthanide‐doped upconversion nanoparticles (UCNPs) and a photosensitizer of methylene blue (MB) in silica nanoparticles, and obtained a smart antimicrobial nanohybrid (UCMB‐LYZ‐HP) after coating with lysozyme (LYZ) and the mixture of hyaluronic acid (HA) and poly‐L‐lysine (PLL) (termed HP) on the surface of silica nanoparticles (**Figure** **4**a).^[^ ^65^ ^]^ UCMB‐LYZ‐HP quickly adsorbed onto the surface of bacteria through electrostatic interaction, and produced a large amount of ROS to destroy bacteria upon laser irradiation. In addition, the HP layer of UCMB‐LYZ‐HP was degraded by the bacteria‐secreted HAase, resulting in the massive release of LYZ and continuous killing of bacteria. Experiments showed that UCMB‐LYZ‐HP enabled effective treatment of MRSA infections in deep tissues (5 mm thick) without causing any side effects.

In addition to directly coupling photosensitizers to nanocarriers, nanonization of the photosensitizer is also an effective method to prepare antimicrobial nPDT. Wang et al. synthesized a self‐assembled porphyrin nano‐photosensitizer with strong NO adsorption capability (ZnTPyP@NO).^[^ ^66^ ^]^ This nano‐photosensitizer was synthesized by a confined noncovalent self‐assembly process using zinc meso‐tetra (4‐pyridyl) porphyrin (ZnTPyP), and it could further absorb NO to coordinate with the central Zn ion, thus forming ZnTPyP@NO nanoparticles (Figure 4b). This ZnTPyP@NO nano‐photosensitizer exhibited dual capabilities of ROS generation and NO release, and achieved synergistic antimicrobial performance. Recently, Deng et al. loaded a porphyrin‐based metal–organic framework (pMOF) coated with human serum albumin to prepare multicomponent antimicrobial photodynamic nanoplatforms (MMNPs) that can effectively eradicate bacterial biofilms.^[^ ^67^ ^]^ In an acidic biofilm environment, the MMNPs were effectively degraded to release the positively charged pMOFs that had strong bacterial adhesion and biofilm permeability and produced ROS to eradicate biofilms upon NIR light irradiation. By fully utilizing the unique pathological microenvironment of biofilms to achieve targeted and enhanced ROS production, MMNPs provide an effective therapeutic modality for microbial biofilm infections. These nano‐photosensitizers, with a high absorption coefficient and photostability, have become the focus in the field of antimicrobial nPDT, exhibiting great potential for future bacterial infection treatment.

### 3.3 Nano‐Photothermal Therapy (nPTT)

As a classic physical therapeutic modality, photothermal therapy (PTT) involving hyperthermic damage to targeted cells with a combination of photothermal agents and light stimulation, has been regarded as an alternative strategy to combat bacterial infections.^[^ ^68^ ^]^ In the PTT process, photothermal agents can absorb light of an appropriate wavelength upon irradiation, and can then transfer the energy into heat by non‐radiative relaxation in the surrounding environment.^[^ ^69^ ^]^ The generated hyperthermal effect causes irreparable damage to the structure of bacteria as well as biofilms, resulting in efficient bacterial elimination without causing resistance.^[^ ^70^ ^]^ With the advancement of nanotechnology, a slew of well‐designed antimicrobial nPTT systems have been developed either by coupling conventional photothermal agents with nanomaterials to improve their photostability and pharmacokinetics, or by synthesizing and exploiting novel nanoparticles with higher photothermal conversion efficiency.^[^ ^71^ ^]^

**Figure 5:**
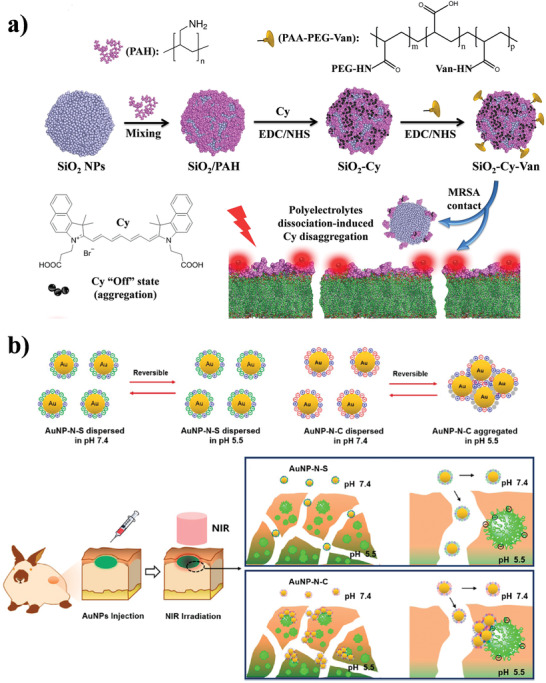
Antimicrobial nPTT. a) Conceptual illustration of the SiO_2_‐Cy‐Van nanoprobe for MRSA‐activated NIR fluorescence imaging and nPTT. Reproduced with permission.^[^ ^73^ ^]^ Copyright 2017, American Chemical Society. b) Schematic illustration of surface‐adaptive gold nanoparticles for effective adherence and localized photothermal ablation of the MRSA biofilm. Reproduced with permission.^[^ ^77^ ^]^ Copyright 2017, American Chemical Society.

Organic dyes that can generate heat in the energy dissipation process after NIR light excitation offer a good platform for nPTT. Among the photothermal organic dyes, cypate is one of the representatives given its high photothermal conversion efficiency as well as NIR fluorescence and outstanding biocompatibility; it has been approved by the US Food and Drug Administration for in vivo applications.^[^ ^72^ ^]^ By exploiting the distinctive properties of cypate, our group designed activatable theranostic nanoprobes (SiO_2_‐Cy‐Van) for NIR fluorescence imaging and nPTT of MRSA infections based on silica nanoparticles coated with vancomycin‐modified polyelectrolyte‐cypate complexes (**Figure** **5**a).^[^ ^73^ ^]^ SiO_2_‐Cy‐Van exhibited an obvious cypate‐concentration‐dependent photothermal effect along with outstanding photothermal stability, and was non‐fluorescent because of the aggregation of hydrophobic cypate on silica nanoparticles to induce ground‐state quenching. Interestingly, the fluorescence of SiO_2_‐Cy‐Van was recovered in the presence of MRSA due to the bacteria‐responsive dissociation of the polyelectrolyte layer from silica nanoparticles, which changed the state of cypate from aggregation to disaggregation. Experimental results demonstrated that SiO_2_‐Cy‐Van possessed enhanced accumulation and long‐term retention (up to 16 days) at the MRSA‐infected site, and achieved highly sensitive (10^5^ colony‐forming units) in vivo diagnosis and efficient nPTT of the MRSA infection. Recently, we presented a cypate and proline (procollagen component)‐loaded pH‐switchable antimicrobial hydrogel with nanofiber networks for biofilm eradication and for rescuing stalled healing in chronic wounds, based on the self‐assembly of a designed octapeptide (IKFQFHFD) at neutral pH.^[^ ^74^ ^]^ This hydrogel exhibited pH (pH ≈ 5.5)‐dependent nanofiber network destabilization and activated release of loaded cypate and proline, achieving targeted photothermal elimination of biofilm and accelerating the subsequent wound healing cascade. The in vivo results demonstrated that this antimicrobial hydrogel with nanofiber networks enabled complete healing of MRSA biofilm‐infected wounds in diabetic mice within 20 days, thus showing great potential for chronic wound management as an antimicrobial dressing.

With the development of the localized surface plasmon resonance, Au nanoparticles have become widely used as photothermal nanoagents for antimicrobial nPTT. He et al. developed a biogenic Au NP/polyethylene glycol (PEG)‐based coating for the photothermal eradication of bacteria.^[^ ^75^ ^]^ By exploiting the photothermal activity of gold nanomaterials, this Au NP/PEG‐based coating exhibited remarkable antimicrobial properties under NIR irradiation both in vitro and in vivo. However, PTT has a serious problem that cannot be ignored; the non‐local heat generated by the photothermal agent often causes great damage to the surrounding healthy tissues while thermally killing bacteria.^[^ ^76^ ^]^ To overcome this drawback, Hu et al. prepared surface‐adaptive Au nanoparticles that showed a fast acidic pH‐responsive transition from negative charge to positive charge.^[^ ^77^ ^]^ This unique charge transition characteristic made the surface‐adaptive Au nanoparticles disperse well in healthy tissues (pH ≈ 7.4), while quickly exhibiting strong adherence to negatively charged bacterial biofilms (pH ≈ 5.5) to form aggregates. Under NIR light irradiation, the aggregated Au nanoparticles within the biofilm enabled the enhanced photothermal eradication of MRSA biofilm, while no damage was inflicted on the surrounding healthy tissues due to the negligible photothermal effect of the dispersed Au nanoparticles (Figure 5b). This work cleverly utilizes the difference in pH between the biofilm and healthy tissues, and provides a reference for localized photothermal elimination of bacteria to avoid damage to healthy tissues.

Besides gold nanoparticles, some semiconductor nanomaterials, such as n‐type semiconductors of CuS nanoparticles (CuS NPs), have gained recognition as photothermal agents for antimicrobial nPTT due to their high photothermal stability and low cost. Recently, Zhou et al. combined PEG‐functionalized CuS NPs with a 3D network of oxidized dextran (ODex) to develop an injectable antimicrobial nPTT hydrogel system.^[^ ^78^ ^]^ The 3D porous structure and hydrophilicity of the hydrogel resulted in a wound with a sustainable moist environment, and the CuS nanoparticles endowed the hydrogel with excellent photothermal performance under NIR laser irradiation to achieve broad‐spectrum bacterial elimination. In addition, conducting polymers have been employed as antimicrobial nPTT.^[^ ^79^ ^]^ Yan et al. constructed a pH‐switchable nanoplatform (PLNP@PANI‐GCS) for precise nPTT of bacterial infections, by grafting the conducting polymers of polyaniline (PANI) and glycol chitosan (GCS) onto the surface of persistent luminescence nanoparticles (PLNPs).^[^ ^80^ ^]^ In the bacteria‐infected acid microenvironment, the number of PLNP@PANI‐GCS amine groups significantly increased to promote the conversion of negative charge to positive charge, achieve bacterial capture, and ensure the precise heating of bacteria. Moreover, some typical 2D nanomaterials with high‐efficiency photothermal conversion capability, such as GO and molybdenum disulfide (MoS_2_), have been utilized for antimicrobial nPTT.^[^ ^81^ ^]^

## 4 Magnetic Stimulation‐Based NPAS

Magnetic sterilization involving the use of magnetic fields, is a classic physical antimicrobial method with the advantages of controllable operation, low cost, and broad antibacterial effects.^[^ ^82^ ^]^ Magnetic sterilization has been widely used in the food industry since the flavor and quality of food are usually unaffected by magnetic field treatment.^[^ ^83^ ^]^ The induced current within magnetic fields that can denature bacterial proteins and cause structural damage is an important mechanism of magnetic sterilization.^[^ ^84^ ^]^ In addition, it has been reported that magnetic fields can affect the adhesion of bacteria, which is believed to be another mechanism of magnetic sterilization.^[^ ^85^ ^]^ With the development of nanotechnology, various magnetic nanoparticles (MNPs) with unique physical properties (e.g., magnetothermal effect and magnetic resonance imaging), have been prepared and employed to develop magnetic sterilization techniques with novel bactericidal mechanisms. In addition, modified MNPs exhibit bacterial recognition and capture efficiency and can be easily controlled and recycled by an external magnetic field, which can be fully exploited to achieve enhanced antimicrobial performance.^[^ ^86^ ^]^

**Figure 6:**
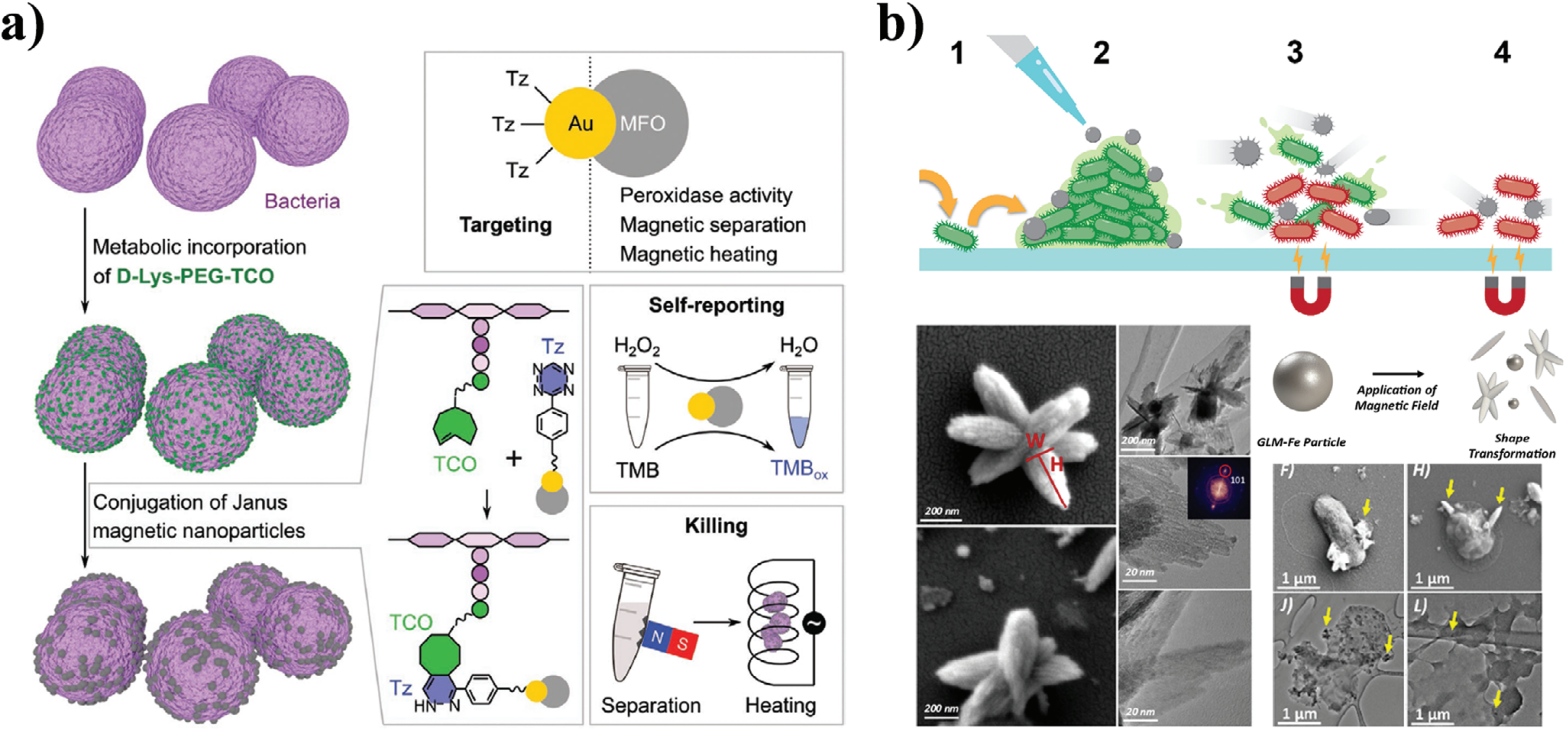
Magnetic stimulation‐based NPAS. a) Schematic illustration of self‐reporting Janus magnetic nanoparticles of Au/MFO for metabolic labeling‐mediated targeting and magnetothermal killing of bacteria. Reproduced with permission.^[^ ^92^ ^]^ Copyright 2021, Wiley‐VCH. b) Magnetic‐activated antimicrobial liquid metals for biofilm elimination. Reproduced with permission.^[^ ^93^ ^]^ Copyright 2020, American Chemical Society.

MNPs can convert magnetic energy to heat through Neel relaxation and Brownian relaxation under the stimulation of alternating magnetic fields, thus offering a novel magnetic sterilization mechanism via the magnetothermal effect of MNPs. The heat generated by MNPs depends on the particle size and relaxation time.^[^ ^87^ ^]^ When the particles are small and the relaxation time is comparable to the frequency, a large amount of heat is generated.^[^ ^88^ ^]^ Fe_3_O_4_ NPs, the most widely used magnetic nanoparticles with good biocompatibility and safety, are effective heating materials for local hyperthermia under an alternating magnetic field.^[^ ^89^ ^]^ By embedding Fe_3_O_4_ NPs in ZnO, Singh et al. synthesized an Fe_3_O_4_‐ZnO nanocomposite, and experimental results demonstrated that the Fe_3_O_4_‐ZnO nanocomposite showed excellent heat‐activated killing of bacteria under an alternating magnetic field even at low concentrations.^[^ ^90^ ^]^ In addition, Janus nanoparticles composed of two or more different properties have provided an integrated treatment platform for functionalized magnetothermal therapy.^[^ ^91^ ^]^ Hou et al. designed Au/MnFe_2_O_4_ (Au/MFO) Janus magnetic nanoparticles to target bacteria and carry out integrated self‐reporting and magnetothermal killing of bacteria (**Figure** **6**a).^[^ ^92^ ^]^ In the Au/MFO Janus magnetic nanoparticles, the Au component was functionalized with tetrazine (Tz) to target the trans‐cyclooctene (TCO) group of D‐lysine anchored on the bacterial cell wall via metabolic incorporation, while the MFO component was responsible for bacterial self‐reporting and magnetothermal elimination.

In addition to the magnetothermal effect, non‐magnetothermal magnetic antimicrobial mechanisms have been reported. Recently, Elbourne et al. studied the antibacterial activity of magnetic galinstan‐based liquid metal nanoparticles (GLM‐Fe).^[^ ^93^ ^]^ The GLM‐Fe had a regular spherical structure, and did not exhibit inherent bacterial toxicity in the absence of a magnetic field. Surprisingly, the GLM‐FE with regular spherical structure changed their morphology from large spheres to smaller 3D extruded particles with sharp edges when exposed to a low‐intensity rotating magnetic field. When introduced into bacterial biofilms, the movement of GLM‐Fe resulting from magnetic field exposure, coupled with the presence of nano‐sharp edges, enabled efficient elimination of bacteria within mature biofilms via a physical mechanism involving bacterial structure disruption (Figure 6b). The magnetically activated GLM‐Fe exhibited broad‐spectrum antimicrobial activity against gram‐negative *P. aeruginosa* and gram‐positive *S. aureus* biofilms without causing resistance, providing a proof‐of‐concept for the non‐magnetothermal magnetic antimicrobial mechanism.

## 5 Acoustic Stimulation‐Based NPAS

Acoustic stimulation has been reported to exhibit antibacterial performance based on the cavitation effect.^[^ ^94^ ^]^ In particular, ultrasound can cause the local pressure of the tissue fluid to decrease, thereby forming bubbles or cavities around the tiny gas cores. These small bubbles grow and close more strongly as the sound pressure increases and finally burst, generating shear forces and free radicals to destroy the bacterial structure. Sonodynamic therapy (SDT) is one of the most used strategies in ultrasonic sterilization.^[^ ^95^ ^]^ Similar to the principle of the photodynamic process, SDT uses ultrasound to excite the sonosensitizers and generate electron transfer that can react with water and oxygen and produce antimicrobial ROS. Compared with light stimulation, ultrasound stimulation has better tissue penetration and targeting capacities that can reduce damage to healthy host tissues.^[^ ^96^ ^]^ However, as with photosensitizers, sonosensitizers also suffer from poor hydrophilicity and stability, greatly weakening the antimicrobial performance of SDT. By employing nanomaterials as carriers of traditional sonosensitizers to improve their hydrophilicity and stability, or by synthesizing and exploiting novel nanoparticles with sonodynamic effects, various antimicrobial nano‐sonodynamic systems with reinforced bactericidal performance have been developed to combat bacterial infections.

**Figure 7:**
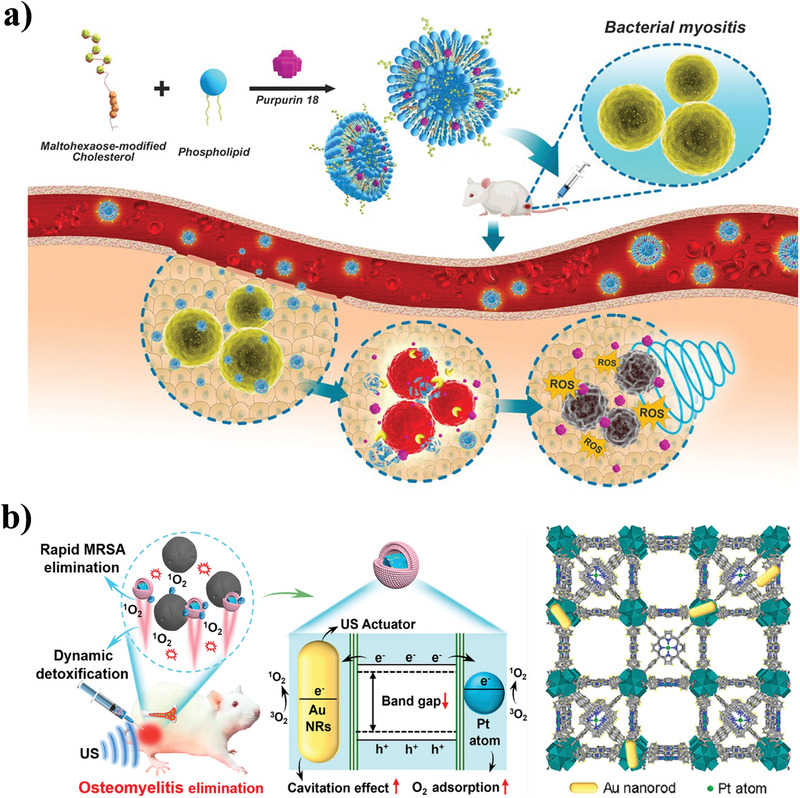
Acoustic stimulation‐based NPAS. a) Schematic illustration of sonodynamic MLP18 nanoliposome for multidrug‐resistant bacterial infections. Reproduced with permission.^[^ ^97^ ^]^ Copyright 2019, American Chemical Society. b) Schematic illustration of ultrasound‐activated single‐atom catalyst of RBC‐HNTM‐Pt@Au for SDT of MRSA‐infected osteomyelitis. Reproduced with permission.^[^ ^100^ ^]^ Copyright 2021, American Chemical Society.

The combination of nanomaterials and traditional sonosensitizers can improve the stability and targeting capacity of sonosensitizers to enhance the antimicrobial effect of SDT. Pang et al. designed a bacteria‐responsive purpurin 18 sonosensitizer‐loaded nanoliposomes (MLP18) for SDT of multidrug‐resistant (MDR) bacterial infections (**Figure** **7**a).^[^ ^97^ ^]^ MLP 18 was prepared based on the assembly of maltohexaose‐modified cholesterol and 1,2 dioctadecanoyl‐sn‐glycero‐3‐phospho‐rac (1′‐glycerol) (DSPG)‐containing lipid compositions. Maltohexaose‐modified cholesterol enabled MLP18 to target the bacterial infection site through the bacteria‐specific maltodextrin transport pathway, while the DSPG‐containing lipid component was sensitive to the bacteria‐secreted phospholipase A2 to activate the release and internalization of purpurin 18. The massive release and internalization of purpurin 18 resulted in effective sonodynamic elimination of MDR bacteria. Furthermore, by loading the sonosensitizer tetrakis (4‐sulfonylphenyl) porphyrin (TPPS) into the nanovesicles genetically displaying neutralizing monoclonal antibody of MEDI4893 against the MRSA toxin on their surface, Pang et al. prepared monoclonal antibody pilot nanovesicles (ANVs) with both toxin neutralization and sonodynamic antibacterial effects.^[^ ^98^ ^]^ In these ANVs, MEDI4893 effectively captured MRSA bacteria and neutralized their virulence, while the sonosensitizer was activated by ultrasound to produce ROS to kill bacteria and assist bacterial clearance by MEDI4893. In addition, the ANVs also allowed precise optical diagnosis of MRSA infection due to the highly specific antibody‐ligand interaction and inherent luminescent features of TPPS sonosensitizers.

As a minimally invasive method, the excellent penetrability of ultrasound makes SDT an excellent candidate for the treatment of deep infections. However, given the low utilization rate of traditional sonosensitizers in deep tissues, enhancing the efficiency of SDT has become the focus of research.^[^ ^99^ ^]^ Yu et al. developed an ultrasound‐activated single‐atom catalyst (RBC‐HNTM‐Pt@Au) consisting of an Au nanorod (Au NR)‐actuated Pt single‐atom‐doped porphyrin metal‐organic framework (HNTM‐Pt@Au) and red blood cell (RBC) membrane to treat osteomyelitis caused by MRSA (Figure 7b).^[^ ^100^ ^]^ Under the action of ultrasound, the Au NRs and monoatomic Pt in the RBC‐HNTM‐Pt@Au improved the electron transfer efficiency and weakened the recombination efficiency of electron‐hole pairs, thereby significantly enhancing the sonocatalytic ability of the sonosensitizer of porphyrin to achieve antimicrobial SDT of deep tissue infections related to osteomyelitis. Meanwhile, the Au NRs were driven by ultrasound to directionally propel the movement of RBC‐HNTM‐Pt@Au and neutralize the toxins secreted by RBCs, achieving a reinforced antimicrobial therapeutic effect. Moreover, researchers are committed to the exploration and development of new sonosensitizers. Wu et al. developed a new type of sonosensitizer (Au@BTO) for high‐efficiency antimicrobial SDT based on ferroelectric ceramic barium titanate (BTO) nanotubes with a Schottky junction modified by Au nanoparticles.^[^ ^101^ ^]^ Ultrasound, as an exogenous mechanical wave, triggered the piezotronic effect of Au@BTO to facilitate the separation and migration of charge carriers at the piezoelectric/metal surface. These polarized charges and carriers further reacted with the surrounding oxygen and water to generate ROS for antimicrobial SDT. In vitro and in vivo experiments demonstrated that Au@BTO exhibited broad‐spectrum bactericidal activity and significantly promoted wound healing under ultrasound irradiation.

## 6 Electrical Stimulation‐Based NPAS

It is well known that electrons are transmitted through proteins of the respiratory chain in organisms, which generates energy to maintain the metabolism and physiological activities of cells.^[^ ^102^ ^]^ Compared with eukaryotes, whose respiratory chain proteins are located in the mitochondria, the proteins of the respiratory chain of bacteria are located on the surface of the cell membrane and are much more sensitive to external electrical disturbances. Research shows that electrical stimulation can effectively disrupt the electron transport of the bacterial respiratory chain, and cause a burst of respiratory byproducts of ROS, leading to the death of bacterial cells.^[^ ^103^ ^]^ As a superior physical antimicrobial modality, electrical stimulation does not cause bacterial resistance and has a bactericidal effect on MDR bacteria. Recently, advancements in nanotechnology have rendered it possible to miniaturize electronic nanosystems and stimulate more personalized and convenient medical antimicrobial strategies.^[^ ^104^ ^]^

**Figure 8:**
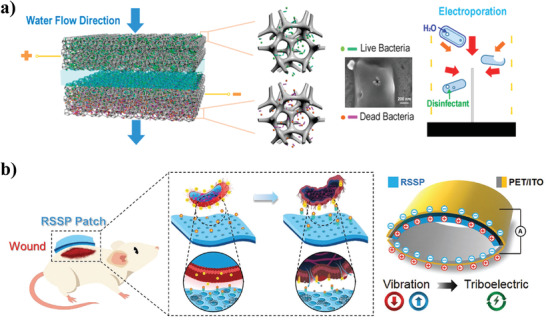
Electrical stimulation‐based NPAS. a) Conducting nano‐sponge electroporation for bacterial disinfection in water. Reproduced with permission.^[^ ^107^ ^]^ Copyright 2013, American Chemical Society. b) Schematic illustration of RSSP‐TENG antimicrobial patch for wound disinfection. Reproduced with permission.^[^ ^109^ ^]^ Copyright 2018, Wiley‐VCH.

As a common transfection method, electroporation with a strong electric field has been demonstrated to destroy bacterial cell membranes. When bacterial cells are exposed to a strong electric field, the transmembrane voltage induced by the electric field promotes the formation of pores in the lipid bilayer, and the strong electric field causes irreversible membrane damage and leads to bacterial death. Generally, the lethal electroporation threshold is between 10 and 35 kV cm^−1^, which means that a high voltage needs to be applied, which leads to high energy consumption and safety issues.^[^ ^105^ ^]^ To reduce the intensity of the electric field used in electroporation, 1D nanostructured electrodes have been introduced. It was proven that the electric field near the tip of the 1D nanostructured electrodes was enhanced by 3–4 orders of magnitude, thus greatly decreasing the power requirements during electroporation.^[^ ^106^ ^]^ Liu et al. designed a conductive nano‐sponge filter device that could achieve efficient water disinfection by electroporation (**Figure** **8**a).^[^ ^107^ ^]^ The filter was made of a commercial polyurethane sponge modified with 1D carbon nanotubes (CNTs) and silver nanowires (AgNWs). The addition of 1D nanomaterials helped to functionalize the polyurethane sponge as a conductive electrode with sharp nano‐level tips, thereby establishing a strong electric field. By applying a very low external voltage (only a few volts), electroporation occurred to cause irreversible damage to the bacterial cell membrane, and the infiltration of water further removed the electroporated dead bacteria, achieving high‐efficiency disinfection. The experimental results demonstrated that this conductive nano‐sponge electroporation‐based strategy enabled more than 6 log (99.9999%) removal of four model bacteria with a low energy consumption (100 J L^−1^). Furthermore, Yue et al. prepared CuO nanowire (CuO NW)‐silver nanoparticle (Ag NP) co‐modified copper foam, and investigated its performance for water disinfection via electroporation by considering the influence of fluid flow.^[^ ^108^ ^]^ Experiments demonstrated that the CuO NW‐Ag NP composite enhanced the electric field strength, and the obtained copper foam exhibited a high‐efficiency disinfection rate of more than 99.9% with an ultrahigh water flux (1 L min^−1^) at a low voltage, demonstrating significant potential for practical water disinfection.

Apart from nano‐electroporation, triboelectric nanogenerators (TENGs), which use the triboelectricity effect to convert mechanical energy into electrical energy, have been reported and employed for antimicrobial applications. Zhang et al. designed a TENG‐based antimicrobial patch with excellent output power and good biocompatibility using recombinant spider silk protein (RSSP).^[^ ^109^ ^]^ This RSSP‐TENG patch produced effective electrical stimulation to disrupt extracellular electron transport and induce ROS production in bacteria, destroying cell morphology and contributing to bacterial death (Figure 8b). In the fabrication of the RSSP‐TENG patch, water lithography technology was introduced to allow the patch to be easily loaded and doped with nanoparticles, which further enhanced its economic applicability. Recently, Shi et al. proposed a TENG‐based self‐powered strategy to endow titanium implant surfaces with anti‐biofilm activity and osteogenesis.^[^ ^110^ ^]^ The negatively charged implant prevented the adhesion of bacteria, and effectively destroyed mature biofilms by electrical stimulation. Moreover, electrical stimulation produced by TENG significantly promoted the adhesion, osteogenic differentiation, and proliferation of pre‐osteoblasts without side effects. The combination of TENG and nano‐electroporation has also been used for antimicrobial applications. Chiu et al. designed a self‐powered disinfection system composed of multilayered TENG and carbon fiber fabrics (CFF) modified with Au—Te nanowires to harvest biomechanical energy from human motions.^[^ ^111^ ^]^ The TENG was used to disrupt the electronic transport of bacteria, while the Au—Te nanowires grown on CFF with excellent conductivity were designed to enhance the electroporation effect. Due to the hybrid effects of TENG and electroporation, this disinfection system exhibited synergistic antimicrobial performance against gram‐negative *E. coli* and gram‐positive *S. aureus*. Moreover, the electricity generated by this disinfection system can be stored in a capacitor to ensure long‐term antibacterial and disinfection functions via an instantaneous discharging process.

## 7 Multimodal NPAS

Due to its intrinsic physical characteristics and based on the stimulation of mechanical, optical, magnetic, acoustic, and electrical signals, NPAS offers therapeutic modalities for bacterial infections. Individually, all of the NPAS described above present certain application limitations due to their specific physical nature, especially in complex and variable internal environments, and a single NPAS cannot always meet the needs of practical applications.^[^ ^112^ ^]^ Hence, by merging two or more different NPASs to utilize their complementary advantages, multimodal NPAS has emerged to achieve satisfactory therapeutic effects.^[^ ^113^ ^]^ In this section, we briefly introduce several typical examples of multimodal NPAS to illustrate their different advantages and provide references for practical antimicrobial applications.

Targeted bacterial capture is a prerequisite for subsequent bacterial elimination. Although rapid and selective bacterial capture has been achieved using bacteria‐targeting systems, the low sensitivity and capture efficiency of these systems at extremely low bacterial concentrations present challenges, as they limit subsequent bacterial elimination.^[^ ^114^ ^]^ To address this challenge, our group demonstrated a novel strategy for enhanced bacterial capture at trace concentrations (10 colony‐forming units/mL) based on a functionalized 3D nanowire substrate, by utilizing the synergistic effect of nanowire topography and surface chemical modification for bacterial recognition and attachment.^[^ ^115^ ^]^ Furthermore, the enhanced bacterial capture efficiency of this functionalized 3D nanowire substrate enabled on‐demand efficient elimination of bacterial pathogens when combined with controllable phototherapy. Specifically, a human immunoglobulin G (IgG)‐modified 3D nanowire substrate was fabricated and employed for on‐demand photodynamic killing of *S. aureus* using a secondary‐antibody‐bound photosensitizer of rose bengal (RB). Experimental results showed that this nanogeometry/PDT‐based NAPS enabled on‐demand high‐efficiency *S. aureus* elimination upon 550 nm light irradiation. Moreover, the 3D nanowire array could be transferred into a flexible substrate and demonstrated great potential for the treatment of bacterial wound infections as an antimicrobial dressing.^[^ ^116^ ^]^

**Figure 9:**
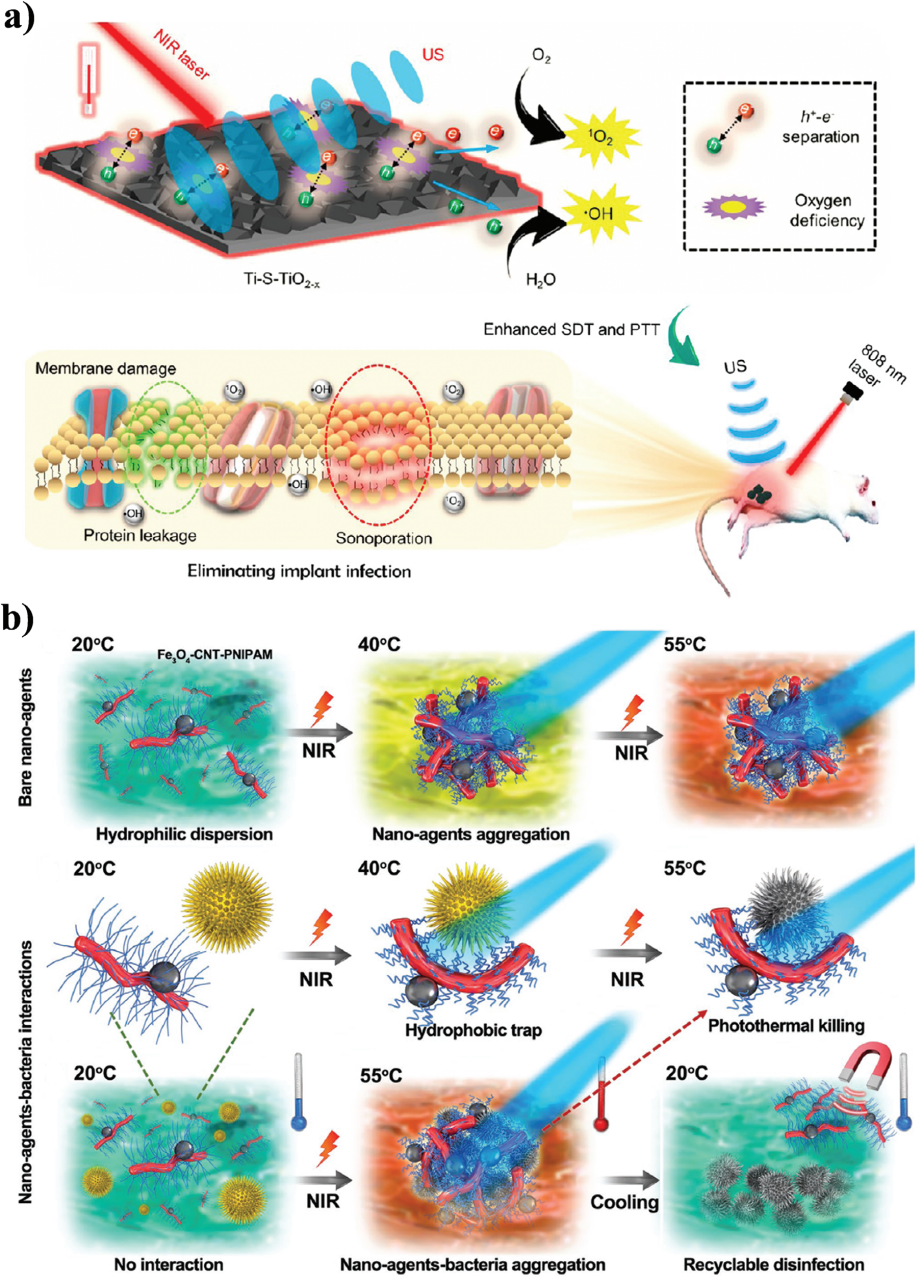
Multimodal NPAS. a) Schematic illustration of Ti‐S‐TiO_2−_ *_x_*‐based photo‐sonotherapy for clinical treatment of bacterial infected bone implants. Reproduced with permission.^[^ ^117^ ^]^ Copyright 2020, American Chemical Society. b) Schematic illustration of Fe_3_O_4_‐CNT‐PNIPAM‐based magnetic/phototherapy for bacterial trapping, ablation, and release to achieve efficient wound disinfection. Reproduced with permission.^[^ ^120^ ^]^ Copyright 2018, Wiley‐VCH.

By exploiting the advantages of optical and acoustic stimulation‐based NPAS, Su et al. designed an oxygen‐deficient sulfur‐doped TiO_2_ layer (Ti‐S‐TiO_2−_ *_x_*) with good sonodynamic, photodynamic, and photothermal effects, which endowed the surface of the titanium implant with antimicrobial ability without introducing an external antibacterial coating (**Figure** **9**a).^[^ ^117^ ^]^ Given its higher specific surface area, Ti‐S‐TiO_2−_ *_x_* promoted the separation of electron‐hole pairs and further improved the sonocatalytic efficiency as well as the degree of oxygen deficiency through sulfur doping, thus enhancing photo‐sonotherapy performance. Under 808 nm laser and US radiation, Ti‐S‐TiO_2−_ *_x_* showed highly effective antimicrobial ability as well as excellent stability and recyclability in a bone implant infection model, accelerating osseointegration in vivo. Moreover, Xu et al. designed multifunctional upconversion nanoparticles (UCNP@SiO_2_‐RB NPs) by enclosing hematoporphyrin monomethyl ether into its yolk‐structured up‐conversion core and covalently linking the photosensitizer of RB on its silica (SiO_2_) shell.^[^ ^118^ ^]^ The UCNP@SiO_2_‐RB NPs possessed combined photodynamic/sonodynamic ability and achieved 100% antimicrobial efficiency against antibiotic‐resistant pathogenic bacteria, showing great potential for clinical synergistic treatment of bacterial infectious diseases.^[^ ^119^ ^]^

MNPs with superparamagnetism are always employed in combination with light‐stimulation‐based NPAS to develop magnetic/phototherapy for antibacterial applications. Yang et al. reported a stimulus‐responsive regulatory mechanism for controlling the trap, ablation, and release activities of pathogenic bacteria, based on the designed nanoagent of CNT‐Fe_3_O_4_ grown with a temperature‐sensitive polymer brush of PNIPAM (Fe_3_O_4_‐CNT‐PNIPAM).^[^ ^120^ ^]^ The Fe_3_O_4_‐CNT‐PNIPAM solution could rise from 19.8 to 54.0 °C within 5 min upon 808 nm NIR laser irradiation, showing good photothermal efficiency. In addition, PNIPAM exhibited a sensitive response to temperature, and changed from hydrophilic to hydrophobic when the temperature increased. Therefore, upon NIR light irradiation, the Fe_3_O_4_‐CNT‐PNIPAM generated heat and formed stable large‐sized aggregates due to the transfer of PNIPAM, which firmly and quickly adhered to the surface of bacteria and biofilms and exhibited high antimicrobial ability in wound infections in mice due to its strong photothermal ability (Figure 9b). Moreover, the superparamagnetic property of Fe_3_O_4_‐CNT‐PNIPAM allowed it to be reused many times to achieve recycling.

Electrical/magnetic therapy based on radiofrequency electromagnetic stimulation (RF‐EMS) has opened a safe and effective path for the development of multimodal NPAS. Recently, Batool et al. designed an antibacterial nanobot (Ab‐nanobot) by covalently conjugating a cell wall‐binding domain (CBD)‐endolysin with an actuator of Fe_3_O_4_/SiO_2_ with a core‐shell nanostructure.^[^ ^121^ ^]^ The CBD‐endolysin modification conferred the Ab‐nanobot‐specific binding and bacteria‐accumulation ability. Under exposure to RF‐EMS signals, Ab‐nanobots generated localized heat and reacted with hydrogen peroxide (a by‐product of cell metabolism) to produce toxic ∙OH. Therefore, Ab‐nanobot specifically captured and eradicated MRSA in vivo with extremely high efficiency (99.9999%) within 20 min in response to medically approved RF‐EMS signals via cell wall damage. This Ab‐nanobot‐based electrical/magnetic therapy would not promote the development of bacterial resistance and therefore offers a nanomedical resistance‐free strategy to combat MDR bacterial infections. In addition, Chaurasia et al. used Fe_3_O_4_ NPs as the core and SiO_2_‐NH_2_ as the shell to form Fe_3_O_4_@SiO_2_‐NH_2_ nanoparticles, which also demonstrated high antibacterial effects when exposed to RF‐EMS signals.^[^ ^122^ ^]^

It is worth noting that the concept of using thermoelectric materials to induce charge separation for ROS generation and thermal catalysis has been proposed and used for air purification and sustainable environmental remediation. Recently, Lin et al. synthesized a homogeneous hexagonal Bi_2_Te_3_ nanosheet thermal catalyst (Bi_2_Te_3_ NPs) and verified its antibacterial performance.^[^ ^123^ ^]^ Given their excellent Seebeck coefficient and high surface area, Bi_2_Te_3_ NPs have been proven to exhibit efficient thermocatalytic activity and disinfection properties. Experiments showed that Bi_2_Te_3_ NPs could catalyze the production of a large amount of hydrogen peroxide and kill 95% of *E. coli* only under a temperature difference of 20 K. Moreover, the charge separation produced by the temperature difference was beneficial for inhibiting electron‐hole recombination, which ensured the sustainable disinfection ability of Bi_2_Te_3_ NPs. By coating Bi_2_Te_3_ NPs on CFF to manufacture a household antibacterial filter (Bi_2_Te_3_@CFF), the experiment demonstrated the long‐term stability and effective antibacterial ability of Bi_2_Te_3_@CFF, which provides effective support for indoor air purification technology.

## 8 Challenges and Perspectives

This review summarizes recent noteworthy progress in NPAS, based on the stimulation of mechanical, optical, magnetic, acoustic, and electrical signals. By combining the booming nanotechnology with traditional physical antibacterial approaches, numerous types of NPAS have been reported and employed to eliminate bacteria, eradicate biofilms, and treat infectious diseases (**Table** **1**). These studies have demonstrated the superior performance of NPAS to destroy pathogenic bacteria without causing resistance and exhibit broad‐spectrum bactericidal activity against MDR bacteria, proposing a new pathway to combat bacterial infections. More significantly, the NPAS can adjust the structure or composition of nanomaterials to respond to a variety of physical stimuli, thereby greatly broadening the boundaries of its biomedical applications.

**Table 1:** A Summary of the listed examples of NPAS

Stimulation	Mechanism	Materials	Bacteria	Application	Ref.
Mechanical	Stretching	bSi	*P. aeruginosa S. aureus B. subtilis* spores *B. subtilis*	Surface sterilization	^[^ ^29^ ^]^
	“Nanoknife” cutting	Graphene nanosheets	*E. coli*	Sterilization	^[^ ^39^ ^]^
Optical	nPCT	TiO_2_/GDY nanofiber	MRSA	Implant sterilization	^[^ ^50^ ^]^
		D‐TiO_2_/Au@UCN	*E. coli* MRSA	Inactivate bacteria	^[^ ^52^ ^]^
		I‐ZnO‐n	*E. coli*	Sterilization	^[^ ^53^ ^]^
		MnO_2_/g‐C_3_N_4_ heterojunction	*S. aureus E. coli*	Sterilization	^[^ ^57^ ^]^
	nPDT	Fe_3_O_4_‐Ce6‐Apt	*E. coli S. aureus*	Blood disinfection	^[^ ^63^ ^]^
		SiO_2_/PAH‐Ce6	MRSA, *E. coli S. aureus*	Inactivate bacteria	^[^ ^64^ ^]^
		UCMB‐LYZ‐HP	MRSA	Inactivate bacteria	^[^ ^65^ ^]^
		ZnTPyP@NO	*E. coli S. aureus*	Inactivate bacteria	^[^ ^66^ ^]^
		MMNPs	*E. coli S. aureus*	Biofilms eradication	^[^ ^67^ ^]^
	nPTT	SiO_2_‐Cy‐Van	MRSA	Inactivate bacteria	^[^ ^73^ ^]^
		IKFQFHFD(cypate)	MRSA	Biofilms eradication & wound healing	^[^ ^74^ ^]^
		Au NPs/PEG‐based coating	*E. coli* MRSA	Anti‐infection medical devices	^[^ ^75^ ^]^
		AuNP‐N‐C	MRSA	Biofilms eradication	^[^ ^77^ ^]^
		CuS hydrogel system	*E. coli S. aureus*	Wound healing	^[^ ^78^ ^]^
		PLNP@PANI‐GCS	*E. coli S. aureus*	Inactivate bacteria	^[^ ^80^ ^]^
Magnetic	Magnetic hyperthermia	Au/MFO Janus magnetic nanoparticles	*S. aureus B. subtilis*	Treatment of skin and urinary tract infections	^[^ ^92^ ^]^
	Magnetic deformation	GLM‐Fe	*P. aeruginosa S. aureus*	Biofilm removal	^[^ ^93^ ^]^
Acoustic	SDT	MLP18	MRSA	Inactivate bacteria	^[^ ^97^ ^]^
		ANVs	MRSA	Inactivate bacteria	^[^ ^98^ ^]^
		RBC‐HNTM‐Pt@Au	MRSA	Treatment of osteomyelitis	^[^ ^100^ ^]^
		Au@BTO	*E. coli S. aureus*	Wound healing	^[^ ^101^ ^]^
Electrical	Electroporation	Nano‐sponge filter device	*Salmonella*	Water disinfection	^[^ ^107^ ^]^
		CuO NW‐Ag NP composite	*E. coli S. aureus*	Water disinfection	^[^ ^108^ ^]^
	Disrupting metabolism	RSSP‐TENG patch	*E. coli S. aureus*	Inactivate bacteria	^[^ ^109^ ^]^
		TENG‐based Ti implant	*E. coli S. aureus*	Orthopedic and dental implants	^[^ ^110^ ^]^
		m‐TENG	*E. coli S. aureus*	Wearable electronics	^[^ ^111^ ^]^
Multimodal	Stretching/nPDT	Functionalized 3D SiNW substrate	*S. aureus*	Sterilization	^[^ ^115^ ^]^
	SDT/nPTT	Ti‐S‐TiO_2‐x_	*Porphyromonas S. aureus*	Implants sterilization	^[^ ^117^ ^]^
	SDT/nPDT	UCNP@SiO_2_‐RB NPs	MRSA *E. coli*	Inactivate bacteria	^[^ ^118^ ^]^
	Magnetic/nPDT	Fe_3_O_4_‐CNT‐PNIPAM	*E. coli S. aureus*	Wound disinfection & implant sterilization	^[^ ^120^ ^]^
	Electromagnetic	Ab‐nanobot	MRSA	Inactivate bacteria	^[^ ^121^ ^]^
	Electromagnetic	Fe_3_O_4_@SiO_2_‐NH_2_ nanoparticles	*E. coli S. aureus*	Inactivate bacteria	^[^ ^122^ ^]^
	Thermoelectric	Bi_2_Te_3_ NPs	*E. coli*	Environmental remediation	^[^ ^123^ ^]^

From bench to bedside, the nascent field of NPAS still faces several challenges and obstacles. The following points summarize these challenges and outline the general considerations for future NPAS designs from our perspective.

1. Toxicity of Nanomaterials: Although most nanomaterials have been reported to be harmless to human cells and tissues, their potential accumulative damage, as well as long‐term cytotoxicity and carcinogenicity, cannot be ignored. Intravenously administered nanomaterials can easily accumulate in the capillary‐rich liver and spleen, increasing oxidative stress and arousing immune effects to cause long‐term biotoxicity. Therefore, effectively reducing the toxicity of nanomaterials has become an urgent challenge in the field of NPAS. From our perspective, there are two promising approaches to improve this situation. One is to further reduce the size of selected nanomaterials in NPAS to decrease their accumulation in the liver and spleen and accelerate their renal metabolism. Another is to fabricate NPAS based on degradable nanomaterials that can be gradually degraded in the physiological microenvironment or under physical stimuli, which not only ensures the antimicrobial performance of NPAS, but also effectively reduces their biotoxicity after treatment.
2. Clinical Transformation: Physical antimicrobial approaches have been widely used in food sterilization and domestic water treatment for a long time, but their application in the medical field has not been fully developed, particularly in clinical treatment. The vigorous development of physical stimuli‐activated nanomaterials has greatly expanded the application fields of traditional physical antimicrobial methods, rendering them suitable candidates for clinical applications. However, the physiological environment is more complicated than experimental simulations; it can have various effects on the structure and antibacterial effect of nanomaterials selected and further greatly affect the antimicrobial performance of NPAS. There is still a long way to go in translating NPAS into commonly used clinical antimicrobials. To address this challenge, more advanced real‐time detection and imaging technologies are expected to help provide researchers with an in‐depth understanding of the changes in the structure‐function relationship of employed nanomaterials and the antimicrobial performance of NAPS at in situ physiological conditions. In addition, the development of more accurate bacteria‐ and biofilm‐targeting NPAS is an inevitable trend in the future to achieve enhanced bacterial eradication efficacy and low potential damage of healthy cells and tissues in the clinic.
3. Integration and Miniaturization: Due to the complex needs of modern clinics, a single NPAS can no longer achieve a satisfactory therapeutic effect. The development of multimodal NPAS is a key objective for the treatment of bacterial infections in the future by utilizing nanomaterials as an excellent toolbox to combine and exploit the complementary advantages of different physical antibacterial modalities. However, multimodal NPAS relying on multiple physical stimuli requires multifunctional external energy devices, which greatly limits their flexibility in practical applications. From our perspective, the rise and miniaturization of energy‐harvesting devices has offered a promising solution for the physical dependence and spatial expansion of multimodal NPAS. These devices can obtain mechanical and thermal energy from the human body and convert them into physical stimuli, thereby providing sustainable external stimulation for multimodal NPAS. Additionally, they can also be integrated to improve the flexibility and convenience of multimodal NPAS. However, these energy‐harvesting devices still feature problems related to their durability, stability, safety, and economic costs. Prospectively, the development of high‐efficiency energy harvesting devices is the objective for multimodal NPAS in the future.

In summary, NPAS provides a promising way to solve antibiotic resistance and treat MDR bacterial infections. We expect this review to help researchers of related disciplines understand the current status of NPAS, and further advance this field by providing novel insights into designing powerful weapons in the strenuous campaign against bacterial infections.

## Conflict of Interest

The authors declare no conflict of interest.
